# Development and Validation of Kompetitive Allele-Specific PCR Assays for Erucic Acid Content in Indian Mustard [*Brassica juncea* (L.) Czern and Coss.]

**DOI:** 10.3389/fpls.2021.738805

**Published:** 2021-12-15

**Authors:** Karanjot Singh Gill, Gurpreet Kaur, Gurdeep Kaur, Jasmeet Kaur, Simarjeet Kaur Sra, Kawalpreet Kaur, Kaur Gurpreet, Meha Sharma, Mitaly Bansal, Parveen Chhuneja, Surinder S. Banga

**Affiliations:** ^1^Department of Plant Breeding and Genetics, Punjab Agricultural University, Ludhiana, India; ^2^School of Agricultural Biotechnology, Punjab Agricultural University, Ludhiana, India

**Keywords:** KASPar, erucic acid, SNPs, *FAE* genes, allotetraploid

## Abstract

*Brassica juncea L.* is the most widely cultivated oilseed crop in Indian subcontinent. Its seeds contain oil with very high concentration of erucic acid (≈50%). Of late, there is increasing emphasis on the development of low erucic acid varieties because of reported association of the consumption of high erucic acid oil with cardiac lipidosis. Erucic acid is synthesized from oleic acid by an elongation process involving two cycles of four sequential steps. Of which, the first step is catalyzed by β-ketoacyl-CoA synthase (KCS) encoded by the fatty acid elongase 1 (*FAE1*) gene in Brassica. Mutations in the coding region of the *FAE1* lead to the loss of KCS activity and consequently a drastic reduction of erucic acid in the seeds. Molecular markers have been developed on the basis of variation available in the coding or promoter region(s) of the *FAE1*. However, majority of these markers are not breeder friendly and are rarely used in the breeding programs. Present studies were planned to develop robust kompetitive allele-specific PCR (KASPar) assays with high throughput and economics of scale. We first cloned and sequenced *FAE1.1* and *FAE1.2* from high and low erucic acid (<2%) genotypes of *B. juncea* (AABB) and its progenitor species, *B. rapa* (AA) and *B. nigra* (BB). Sequence comparisons of *FAE1.1* and *FAE1.2* genes for low and high erucic acid genotypes revealed single nucleotide polymorphisms (SNPs) at 8 and 3 positions. Of these, three SNPs for *FAE1.1* and one SNPs for *FAE1.2* produced missense mutations, leading to amino acid modifications and inactivation of KCS enzyme. We used SNPs at positions 735 and 1,476 for genes *FAE1.1* and *FAE1.2*, respectively, to develop KASPar assays. These markers were validated on a collection of diverse genotypes and a segregating backcross progeny. KASPar assays developed in this study will be useful for marker-assisted breeding, as these can track recessive alleles in their heterozygous state with high reproducibility.

## Key Message

Developed KASPar assays for selection of low erucic acid are high throughput, robust, and codominant capable to track recessive alleles in their heterozygous state.

## Introduction

Indian mustard [*Brassica juncea* (L.) Czern and Coss] is an important source of edible and industrial oil in the Indian subcontinent. Its seeds contain nearly 40% oil on a seed weight basis. Similar to other plants, oil in Indian mustard is a complex mixture of triglycerides (>95%) along with diacylglycerols (<5%), phytosterols, etc. Though mustard oil contains seven major fatty acids, erucic acid, a very long chain fatty acid (C22:1) is the predominant fatty acid contributing more than 50% to the total pool of fatty acids. Such levels are undesirable in view of their perceived role in cardiac lipidosis and fatty deposits in skeletal muscles (Vogtmann et al., 1975; Burrows and Tyrl, 2013). This concern precipitated substantive efforts to genetically reduce the erucic acid content in different rapeseed-mustard crops. Canadian rapeseed breeders were first to develop cultivars with low erucic acid (<2%) in *B*. *napus* and *B. rapa* (Downey and Craig, 1964). Combining low erucic acid trait with low glucosinolate content (<30 μmol/g defatted meal) in the meal subsequently aided the development of canola quality rapeseed cultivars. Similar success was achieved in *B. juncea* with the discovery of low erucic acid (<2%) genotypes (Zem 1 and Zem 2) in Australia (Kirk and Oram, 1981). Only a few varieties and hybrids of canola mustard are currently under cultivation. However, linkage drag continues to be an issue as low erucic acid donors (Zem 1 and Zem 2) and their derivatives are late maturing, small seeded, and possess low seed oil content (Banga et al., 2015). Traditional breeding methods have not been able to eliminate limitations of low oil in the presently available low erucic acid mustard varieties. Transferring the low erucic acid trait into agronomically superior genotypes is difficult, since the trait is double recessive and fatty acid composition is defined by the genotype of the embryo. Each cycle of backcrossing requires one generation of selfing after every backcross. Plants carrying low erucic acid alleles in the self-progenies are selected on the basis of half-seed analysis for the next cycle of recurrent backcrossing to the recurrent parent. A countless number of half seeds must be analyzed in each generation and this procedure is beyond the analytical capacity of most laboratories in mustard-growing countries. Although many rapid screening techniques (e.g., Near-infrared spectroscopy) are now available (Kaur et al., 2016; Sen et al., 2018), these require a large sample size and are not suitable for the analysis of single seeds. The development of functional markers with the ability to differentiate between homozygous and heterozygous states of plants is considered important. It will obviate the wet chemistry analysis for fatty acid profiling and the necessity of selfing after every generation of backcrossing.

The genes involved in the biosynthesis of very long-chain fatty acids (VLCFAs) are well known. Long-chain fatty acids are synthesized *de novo* with two enzyme systems, acetyl-CoA carboxylase (ACCase) and fatty acid synthase (FAS) complex. ACCase catalyzes the formation of malonyl-acyl carrier protein (ACP) (Harwood, 1996), whereas FAS complex catalyzes the elongation of malonyl-ACP into palmitoyl-ACP with seven rounds of four sequential reactions. Elongation of palmitoyl-ACP results in stearoyl-ACP, which is then saturated by plastidal stearoyl-ACP desaturase to produce oleoyl-ACP. The removal of ACP by acyl-ACP thioesterase (FatA/FatB) marks the termination of elongation in plastids and leads to the formation of free fatty acids such as palmitic, stearic, and oleic acid. Most of the free fatty acids are transported to endoplasmic reticulum, where oleic acid is desaturated and elongated to produce polyunsaturated fatty acid and VLCFA. Fatty acid desaturases (FAD) encoded by *FAD* genes catalyze the desaturation reaction (Kunst et al., 1992; Miquel and Browse, 1992), whereas β-ketoacyl-CoA synthase (KCS) catalyzes the first step of two rounds of four sequential chain elongation reactions (James et al., 1995). This enzyme is encoded by the fatty acid elongase 1 (*FAE1*) gene in Brassica. Mutation in the *FAE1* genes impairs the activity of KCS enzyme (Katavic et al., 2002). Orthologs of the *FAE* and *FAD* genes were also reported in different oilseed *Brassica* species (Barker et al., 2007; Wang et al., 2015; Tang et al., 2019; Xue et al., 2020). *B. juncea* (AABB) and *B. napus* (AACC) possess two homologs of *FAE1*.*1* and *FAE1*.*2* genes. These show more than 99% nucleotide identity. High homologies exist between the gene sequences of the gene from *B. juncea*, *B. rapa*, and *B. napus* (Zeng and Cheng, 2014). Four and three single nucleotide polymorphisms (SNPs) in *FAE1.1* and *FAE1.2* differentiated low erucic genotypes from high erucic acid ones in *B. juncea* (Gupta et al., 2004). A total of 26 SNPs caused changes in 13 amino acids in *B. rapa* (Yan et al., 2015). Deletion of two bases in C homolog in *B. napus* produced low erucic acid phenotype (Fourmann et al., 1998). Single nucleotide primer extension (SNuPE) assays have been used to track allelic variation in the *FAE1* gene (Gupta et al., 2004), but these are expensive and tedious to implement. Cleaved amplified polymorphic sequences were also used to characterize *FAE1.1* and *FAE1.2* alleles in *B. juncea* (Saini et al., 2016), but CAPS markers are dominant and these fail to identify recessive alleles in heterozygous state. Recently, Saini et al. (2019) have reported PCR markers based on the sequence variation in the promoter regions of the *FAE1*. These are gel-based markers. A marker-assisted breeding program requires simple but robust molecular assays that benefit from economies of scale. Kompetitive allele-specific PCR (KASPar) genotyping assay is considered as an ideal approach to hasten the breeding processes through rapid genotyping of thousands of samples at very low error rates (Chen et al., 2010; Kumpatla et al., 2012). In this study, we report the development of KASPar assay for high-throughput genotyping of erucic acid content. These allele-specific assays were developed on the basis of SNPs at positions 735 and 1,476 from A and B homologs of *FAE1.1* and *FAE1.2*. These SNPs were identified on the basis of Sanger sequencing of cloned *FAE1.1* and *FAE1.2* genes from both the high and low erucic acid accessions of *B. juncea* and the progenitor species: *B. rapa* and *B. nigra*. All these sequences have been submitted to the National Center for Biotechnology Information (NCBI) and included in Supplementary File 1. The practical utility of the designed KASPar assays was established by validating these in a germplasm collection and a segregating backcross progeny varying for erucic acid contents.

## Materials and Methods

### Plant Material

A total of 29 pure lines of *B. juncea* (25) and inbred lines of its genome donor species, *B. rapa* (2) and *B. nigra* (2) were selected for this study (Table 1 and Supplementary Table 1). Selected genotypes varied for erucic acid content (0–52%) in the oil and represented a broad spectrum of geographic and genetic diversity. The *FAE1* gene was cloned and sequenced from four genotypes of *B. juncea*, two of *B. rapa* and one of *B. nigra*. A backcross progeny 1 (BC_1_) was developed from a cross of PBR357/RLC3//PBR357 [(High × Low) × High erucic genotype(s)] through hand pollinations. It was raised under field conditions and used to validate KASPar markers in segregating generation.

**TABLE 1:** Diverse germplasm lines of *B. juncea*, *B. rapa*, and *B. nigra*.

Genotype	Origin	Erucic acid (%)	Genotype	Origin	Erucic acid (%)
***B. rapa* (A genome)**					
QR 2#	Indian	0.2	TL 17 #	Indian	42.6
***B. nigra* (B genome)**					
U.P#	Indian	44.5	CN 113799	–	42.0
***B. juncea* (AB genomes)**					
DRMRJ 31	Indian	51.3	ALM 115	Indian	1.8
NRCDR 02	Indian	52.3	CBJ 001	Chinese	1.8
PBR 91#	Indian	45.5	EC 597325	Sweden	0.8
PBR 210	Indian	44.2	ELM 303	Indian	1.8
PBR 357	Indian	45.5	HEERA	European	2.0
PBR 378	Indian	45.0	JLM 102	Indian	1.7
Pusa Bold	Indian	45.0	JM 06013	Australian	1.5
RH 0749	Indian	52.1	PDZ 1	Indian	1.2
RH 8812	Indian	51.8	PM 24	Indian	0.8
RL 1359	Indian	47.9	PM 30	Indian	0.8
RLM 619	Indian	45.2	RLC 1	Indian	1.4
Donskaja	European	21.0	RLC 2	Indian	1.8
–	–	–	RLC 3#	Indian	0.8

*#Genotypes used for sequencing.*

### Fatty Acid Profiling

Fatty acid profile was determined through gas chromatography by following standard protocols (Appelqvist, 1968). For this fatty acid methyl esters (FAMEs) were injected in a fused silica capillary CP-SIL 88 column (50 mm × 0.25 mm id) fitted on gas chromatograph (Varian CP-3800, United States). Temperatures of oven, injector, and Flame ionisation detector (FID) were maintained at 200, 230, and 250^°^C, respectively. The peak identity of different fatty acids was determined by analyzing the FAME mix (Supelco Incorporation, United States) as a reference under similar conditions. The relative area percentage was used for estimating the identified fatty acids.

### Half-Seed Technique for Non-destructive Determination of Fatty Acids

In *Brassica*, embryo genotype determines the composition of fatty acids. This mode of inheritance allows non-destructive estimation of fatty acids at a single seed level. *Brassica* seeds possess conduplicate cotyledons, where the inner cotyledon is attached with germ, and the outer cotyledon covers the inner one. The outer cotyledon was used for ascertaining the fatty acid composition and the inner one was retained for raising into adult plant. To follow half-seed method, the seeds were placed on moistened filter paper in petriplates at 25 ± 2^°^C under dark conditions. The swollen seeds were taken out after 24 h and the outer cotyledon of each seed was separated for estimating the fatty acid composition as per the procedure described earlier.

### Cloning and Sequencing of *FAE1.1* and *FAE1.2*

Full-length *FAE1.1* and *FAE1.2* genes were isolated and cloned separately from high and low erucic acid genotypes of *B. juncea* along with its progenitors *B. rapa* and *B. nigra*. Gene-specific primers for *FAE1.1* and *FAE1.2* were designed by using previously available sequence information (NCBI Gene Accession nos. AJ558197.1 and AJ558198.1). Primers *FAE1.1* AR (forward-TGACGTCATAGTGTTAGGCGT and reverse TTTGGCACCTTTCATCGGAC) and *FAE1.2* BR (forward-ACGAAAGAGAGCAAACATCATTT and reverse-CGAC*A*3 ACACACTGAGCAAT) were got synthesized from IDT, Germany. Thymine/Adenine (TA) cloning kit was used for cloning the amplicons in pGEM-T Easy Vector followed by sequencing with forward and reverse primer of M13. At least five clones from each genotype were sequenced by Sanger dideoxy chain termination method on capillary electrophoresis system (ABI 3730XL, Applied Biosystems, United States). Sanger sequencing was outsourced to the Eurofins Genomics India Pvt. Ltd, India. Vector contamination was removed through VecScreen tool^1^. The consensus sequences were obtained through the Geneious software (Kearse et al., 2012).

### Identification of Single Nucleotide Polymorphisms

Differentiating SNPs were identified by aligning the sequences of *FAE1.1* and *FAE1.2* genes isolated from high erucic and low erucic acid accessions of *B. juncea* and its progenitor species by sequence alignment tool of the Geneious software.

### Development of Kompetitive Allele-Specific PCR Assays

Gene-specific KASPar assays were manually designed for *FAE1.1* and *FAE1.2*. SNPs at positions 735 and 1,476 for *FAE1.1* and *FAE1.2* were used for the purpose (Table 2). Thermodynamic properties of the designed primers were analyzed by using software Vector NTI version 11.5.3^2^.

**TABLE 2:** List of the kompetitive allele-specific PCR (KASPar) primers.

Gene	Melting temperature	Amplicon size (bp)	Primer	Sequence (5′-3′)
*FAE1.1*	67.9	90	KASP-A-735-FAM	GAAGGTGACCAAGTTCATGCTGATGCTCTCTATGCTCACCACAAGG
	68.2		KASP-A-735-VIC	GAAGGTCGGAGTCAACGGATTGATGCTCTCTATGCTCACCACAAGA
	58.5		KASP-A-735-COM	AGTGCCGTCGTTATAGCCATTGAT
*FAE1.2*	67.6	108	KASP-B-1476-FAM	GAAGGTGACCAAGTTCATGCTACGAACCTCTGACTTGGCTGAATCT
	68.2		KASP-B-1476-VIC	GAAGGTCGGAGTCAACGGATTACGAACCTCTGACTTGGCTGAATCA
	58.5		KASP-B-1476-COM	TGGGTGGGTCTAAGCAATGTCAAG

### Kompetitive Allele-Specific PCR Method

Kompetitive allele-specific PCR reaction mix constituted 2 μl DNA (ng), 1.944 μl KASP reagent (LGC Genomics, Beverly, MA, United States) and primer mix of 0.056 μl. PCR was run initially for 15 min at 94^°^C followed by 10 cycles for 20 s at 94^°^C and 1 min at 65^°^C in touchdown mode with a decrease of 1^°^C in every cycle and finally 30 cycles of 20 s at 94^°^C and for 1 min at 55^°^C. The Applied Biosystems™ QuantStudio™ 12K Flex (Thermo Fisher Scientific, United States) was utilized for carrying out PCR reaction and analyzing the PCR product.

## Results

### Variation for Erucic Acid Content

Brassica *rapa* accessions, QR 2 and TL 17, contained 0.2 and 42.6% erucic acid, respectively, while both the genotypes of *B. nigra* (UP and CN 113799) had high erucic acid. *B. juncea* genotypes from India varied between 0.8 and 52.3% for erucic acid in the oil. Erucic acid content of introduced mustard genotypes ranged between 0.8 and 21% (Table 1). EC 597325 and CBJ 001, of Australian and Chinese origin, respectively, exhibited low erucic acid (<2%) in the oil. The East European genotype Donskaja had intermediate erucic acid content.

### Characterization of *FAE1.1*

Sequence size of *FAE1.1* from *B. rapa* cv. TL 17 (high erucic acid) and QR 2 (low erucic acid genotype) was 1,521 bp. The gene(s) comprised a single exon without any intron in *FAE1.1* from both the high and low erucic acid (Supplementary Figure 1). Sequence alignment of *FAE1.1* indicated high similarity (97%). Nucleotide variations were recorded at eight positions (Supplementary Figure 2). These variations manifested into seven transitions and one transversions. A total of 506 amino acids were encoded by messenger RNA (mRNA) of *FAE1.1* gene. Amino acid sequence comparison revealed that amino acid changes at three positions (Supplementary Figure 2). Four SNPs at positions 591, 735, 968, and 1,265 caused transition types of changes and were common across the available database. KASPar primers were designed only at position 735.

### Characterization of *FAE1.2*

Sequence of *B. nigra* genotype, UP with high erucic acid content, was compared with the B homolog from mustard genotype, RLC3 with low erucic acid. *FAE1.2* gene had a sequence length of 1,521 bp with one exon (Supplementary Figure 3). Sequence alignment revealed high homology (99%) between *FAE1.2* gene sequences isolated from *B. nigra* and *B. juncea*. Nucleotide changes were identified only at three positions. These variations were indicative of two transitions and one transversion (Supplementary Figure 4). Translated mRNA sequence of *FAE1.2* encoded 506 amino acids. A comparison of mRNA amino acid sequences of high/low erucic acid *Brassica* genotypes indicated changes only at single position as compared to the changes at three positions for the nucleotide sequence of the gene (Supplementary Figure 4). Gene sequences of *FAE1.2* deciphered in our studies were also aligned with the corresponding sequences available in public domain (NCBI: AJ 558198.1). All the three identified SNPs at positions T/C (49), C/T (237), and T/A (1,476) were consensus. SNP at position 1,476 was selected for developing KASPar primer.

### Identification of Single Nucleotide Polymorphisms Differentiating High/Low Erucic Acid Genotypes in *B. juncea*

Four common SNPs distinguishing high/low erucic acid *FAE1.1* gene were observed in *B. rapa* and *B. juncea* gene sequences. Likewise, three SNPs for high/low erucic acid identified for gene *FAE1.2* in *B. nigra* were also present in *B. juncea*. These SNPs were common across the ploidy and present in all the test germplasm, irrespective of geographic and genetic origin (Table 3 and Supplementary Figure 5).

**TABLE 3:** Single nucleotide polymorphisms (SNPs) differentiating low/high erucic acid (*FAE1.1* and *FAE1.2*) in *Brassica* species.

		SNP Position						
		*FAE1.2*			*FAE1.1*			
Genotypes	Erucic acid	49	237	1,476	591	735	968	1,265
***B. rapa***								
TL 17	49.0	C	C	T	G	C	C	T
QR 2	0.2	C	C	T	A	T	T	C
***B. nigra***								
UP	44.5	T	C	T	T	C	T	T
RLC 3 (B genome)	0.8	C	T	A	T	C	T	T
***B. juncea***								
PBR 91	45.5	T	C	T	G	C	C	T
RLC 3	0.8	C	T	A	A	T	T	C

### Development of the Kompetitive Allele-Specific PCR Assays

**FIGURE 1:**
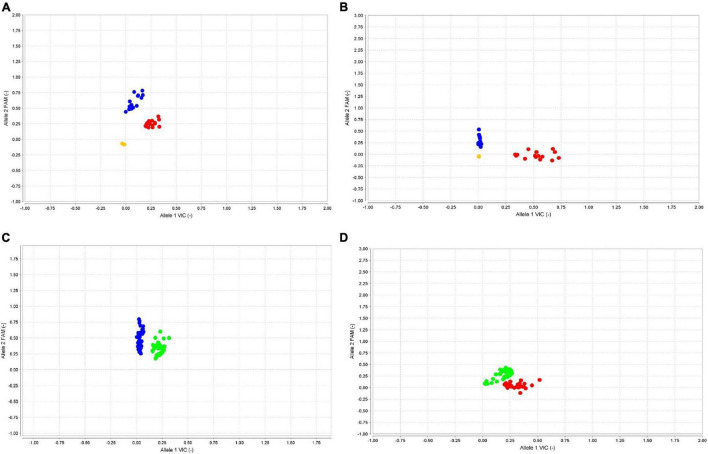
Kompetitive allele-specific PCR (KASPar) genotyping in germplasm lines and backcross population. **(A)** KASPar genotyping of *FAE1.1* gene on pure/inbred lines: genotypes homozygous (*FAE1.1*) for high and low erucic acid clustered to the FAM side (*Y* axis: blue dots) and VIC side (*X* axis: red dots), respectively. **(B)** KASPar genotyping of *FAE1.2* gene on pure/inbred lines: genotypes homozygous for high and low erucic acid (*FAE1.2*) clustered to the VIC side (*X* axis: red dots) and FAM side (*Y* axis: blue dots), respectively. **(C)** KASPar genotyping for *FAE1.1* gene on BC_1_ population. Homozygous genotypes clustered to FAM (*Y* axis: blue dots) whereas heterozygotes clusterd as green dots. **(D)** KASPar genotypes for *FAE1.2* on BC_1_ population. Homozygotes clustered to VIC side (*X* axis: red dots) whereas heterozygotes clustered as green dots.

The KASPar assay for *FAE1.1* was designed for SNPs at position 735 and validated over 29 accessions of *B. rapa*, *B. nigra*, *and B. juncea* (Table 4). Genotypes with low erucic acid formed a cluster distinct from the genotypes with high erucic acid (Figure 1A). Amplification was not observed for *B. nigra* genotypes, indicating the A homolog specificity for the assay developed. KASPar assay specific to the gene *FAE1.2* was designed for SNP at position 1,476. This KASPar assay was also validated on 29 genotypes and formed separate clusters for high vs. low erucic acid genotypes (Table 4 and Figure 1B). *B. rapa* genotypes showed no amplification, confirming the specificity of the assay for B homolog.

**TABLE 4:** Validation of the KASPar assays for erucic acid content in diverse *Brassica* genotypes.

Genotype	Erucic acid (%)	*FAE1.1* (KASPar)	*FAE1.2* (KASPar)
***B. rapa***			
QR 2	0.2	e1e1	NA
TL 17	49.0	E1E1	NA
***B. nigra***			
U.P	44.5	NA	E2E2
CN 113799	42.0	NA	E2E2
***B. juncea***			
DRMRIJ 31	51.3	E1E1	E2E2
NRCDR 02	52.3	E1E1	E2E2
PBR 210	44.2	E1E1	E2E2
PBR 357	45.5	E1E1	E2E2
PBR 378	45.0	E1E1	E2E2
PBR 91	45.5	E1E1	E2E2
Pusa Bold	45.0	E1E1	E2E2
RH 0749	52.1	E1E1	E2E2
RH 8812	51.8	E1E1	E2E2
RL 1359	47.9	E1E1	E2E2
RLM 619	45.2	E1E1	E2E2
Donskaja	21.0	e1e1	E2E2
ALM 115	1.8	e1e1	e2e2
CBJ 001	1.8	e1e1	e2e2
ELM 303	1.8	e1e1	e2e2
EC 597325	0.8	e1e1	e2e2
HEERA	2.0	e1e1	e2e2
JLM 102	1.7	e1e1	e2e2
JM06013	1.5	e1e1	e2e2
PDZ 1	1.2	e1e1	e2e2
PM 24	0.8	e1e1	e2e2
PM 30	0.8	e1e1	e2e2
RLC 1	1.4	e1e1	e2e2
RLC 2	1.8	e1e1	e2e2
RLC 3	0.8	e1e1	e2e2

### Validation of Kompetitive Allele-Specific PCR Assays on BC_1_ Seeds

The KASPar assays were authenticated on BC_1_ seeds, segregating for varied erucic acid content. The seed fatty acid composition was determined by using half-seed method. The outer cotyledon of swollen seeds was harvested for fatty acid analysis, whereas corresponding inner cotyledon with germ portion was seeded to develop into the adult plant. DNA was isolated from first true leaves of plants with known fatty acid composition. The KASPar assays were used for specific amplification of *FAE1.1* and *FAE1.2.* KASP clusters showed equivalence with the segregation expected for a BC_1_ progeny (Figures 1C,D). Four genotypic classes were inferred from the analysis of 75 BC_1_ plants (Table 5). These were E1E1E2E2 (25): E1E1E2e2 (14): E1e1E2E2 (16): E1e1E2e2 (20). Plants with genotype E1E1E2E2 possessed average erucic acid level of 50.35% and plants heterozygous at both the loci (E1e1E2e2) had average erucic acid content of 27.29%. The remaining two genotypic classes, heterozygotes for either of two alleles, E1E1E2e2 and E1e1E2E2 showed erucic acid contents of 39.37 and 35.36%, respectively. E1 allele contributed more than that of the E2 allele. The gene action with an additive effect was indicated for the erucic acid phenotype. Heterozygotes, E1e1E2e2 showed an intermediate phenotype.

**TABLE 5:** Genotyping for *FAE1.1* and *FAE1.2* genes and their association with erucic acid in *B. juncea*.

BC_1_ (No. of samples)	*FAE1.1*	*FAE1.2*	*Range*	Erucic acid (Mean ± SE)
25	E1E1	E2E2	42–53	50.35 ± 0.41
14	E1E1	E2e2	38–41	39.37 ± 0.45
16	E1e1	E2E2	31–37	35.36 ± 0.35
20	E1e1	E2e2	25–30	27.29 ± 0.41
χ2_(1:1:1:1_ *_*p*_*_*value)*_				0.287

*E1/e1, high erucic/low erucic acid allele for FAE1.1 gene; E2/e2, High erucic/low erucic acid allele for FAE1.2 gene.*

## Discussion

Genetic modification of oil quality is now a major breeding objective in mustard. However, most breeding groups are following traditional crop-breeding methods with little application of marker-assisted breeding in India. This is in spite of the availability of many high-density linkage maps and molecular marker systems in the species (Pradhan et al., 2003; Ramchiary et al., 2007; Panjabi et al., 2008; Yadava et al., 2012). KCS enzyme encoded by the *FAE1* is involved in one of the step for the synthesis of erucic acid from oleic acid. The mutation in coding region of the *FAE1* gene impairs its activity and results in drastic reduction of erucic acid (Katavic et al., 2002). Molecular markers, SNuPE (Gupta et al., 2004), and CAPS (Saini et al., 2016) for tracking the allelic variation for the *FAE1* gene, have been developed in *B. juncea*. Recently, a PCR-based marker system has been developed, which exploits the variation in the promoter region of the *FAE1* (Saini et al., 2019). None of these systems allow high throughput and cost-effective genotyping. It is, thus, important to develop breeder friendly and robust marker system.

We independently isolated *FAE1.1* and *FAE1.2* from four genotypes of *B. juncea*, two of *B. rapa*, and one of *B. nigra*. Cloning and gene sequencing allowed us to identify one copy each of *FAE1.1* in *B. rapa* and *FAE1.2* in *B. nigra.* Both the genes (*FAE1.1* and *FAE1.2*) were also detected in allotetraploid *B. juncea* as it has been reported earlier (Das et al., 2002; Gupta et al., 2004; Yan et al., 2015). Comparative sequence analysis has also indicated that the coding sequences (CDSs) from the *FAE1* are highly conserved with high similarity across the tested *Brassica* species. A broader genetic conservation of the gene has also been reported in the related *Brassicaceae* species, *Sinapis alba* (Zeng and Cheng, 2014). The predicted amino acid sequences of *Sinapis alba* have shared high homology with cultivated *Brassica* and *Arabidopsis*. Our studies have also emphasized the role of point/missense mutations in the coding region to inactivate KCS. Differences in the regulatory region or CDS due to SNPs, Insertion/deletions (InDels), and insertion of transposable elements cause dysfunction in the *FAE1* (Roscoe et al., 2001; Katavic et al., 2002; Chiron et al., 2015). In contrast, a deletion of four base pairs in the *FAE1* CDS from *B. napus* has produced a frameshift mutation and led to the formation of non-functional polypeptide with short length (Wu et al., 2008). Serine at 282 positions in the *FAE1* is considered essential for elongase activity in *B. napus* (Katavic et al., 2002). We have also observed transition and transversion types of nucleotide variations at eight positions in *FAE1.1* gene sequences of *B. rapa*. Of these, seven positions have been identified as transition changes and three of which cause amino acid alterations to inactivate KCS. These transition types of alterations have also been reported to cause low erucic acid in *B. rapa* (Wang et al., 2010). A total of 26 SNPs have caused changes in 13 amino acids and consequently loss of activity of KCS has also been reported in *B. rapa* (Yan et al., 2015). Whereas for *B. juncea*, total six nucleotide variations were detected in *FAE1.1* gene and all were of transition types. Comparatively, less nucleotide variations in *FAE1.1* of allotetraploid *B. juncea* were identified as compared to its diploid progenitor, *B. rapa*. Of these variations, four SNPs are common across the ploidy. These SNPs have also been reported earlier by Gupta et al. (2004) in *B. juncea*. For *FAE1.2*, nucleotide variations have occurred at the three positions both in diploid, *B. nigra* and allotetraploid, *B. juncea*. However, only one of these SNPs caused amino acid alteration that led to the loss of function of KCS. SNPs resulting from transition changes in *FAE1.2* gene have also been documented in *B. juncea* (Gupta et al., 2004). Low sequence diversity was recorded for B homologs of the *FAE1* genes in diploid and allotetraploid crop *Brassica* species. SNPs and InDels in gene or genome are being exploited for marker-assisted breeding (Garcés-Claver et al., 2007).

In this study, we planned to develop high throughput and breeder friendly markers capable of discriminating homozygous and heterozygous individuals in the segregating progenies. Effectiveness of the KASPar assays for high-throughput genotyping has already been documented in many crops including *Brassica* (Chhetri et al., 2017; Steele et al., 2018; Chen et al., 2021; Fu et al., 2021). The KASPar assays reported for *FAE1.1* and *FAE1.2* genes in the current communication were validated on large number of diverse genotypes and showed complete associations with erucic acid. High erucic acid *B. juncea* accessions are homozygous dominant for both the genes in contrast to homozygous recessive condition for low erucic acid genotypes as reported earlier (Gupta et al., 2004). Donskaja with intermediate erucic acid level is homozygous recessive for *FAE1.1* and dominant for *FAE1.2*. This observation has explained the intermediate erucic acid levels reported earlier in East European *B. juncea* (Kirk and Hurlstone, 1983). The ability of our present marker system to differentiate between high vs. low erucic acid genotypes from the diverse geographic origins (Australia, India, China, East Europe, and Sweden) indicates its wider applicability. This could be largely due to involvement of Zem 1 and Zem 2 as donors for present low erucic acid cultivars of *B. juncea* (Potts et al., 1999; Bhat et al., 2002). These newly developed KASPar assays are codominant and could discriminate between homozygous and heterozygous states in the BC_1_ progeny investigated. The segregation pattern of these assays is in confirmatory with digenic additive inheritance for erucic acid (Bhat et al., 2002; Gupta et al., 2004). The ability of our marker assays to differentiate homozygous/heterozygous state of alleles in inbred/pure lines and in segregating progenies will expedite the process of marker-assisted selection for low erucic acid in *B. juncea*.

## Conclusion

The KASPar assays were developed on the basis of SNP variations in the coding regions of the *FAE1* genes. These assays are highly genome specific and can discriminate between heterozygous, double recessive, or double dominant states of a genotype. These can be coupled with a background selection to expedite the recovery of the recurrent parent and reduce linkage drag associated with the trait donor parent. Per sample costs are also low and a large number of samples can be processed in 1 day. Developed KASPar assays are single step, high throughput, robust, codominant, and cost-effective than gel-based markers and can be efficiently used for marker-assisted selection of low erucic acid.

## Data Availability Statement

The data set included in this manuscript is appended as Supplementary Material.

## Author Contributions

GK and SB conceived and coordinated the study. GK, SK, KK, KG, and MS developed gene-specific primers and cloned the genes. KSG, GK, GDK, and JK conducted genotyping. GK and KSG looked after the field experiments and the phenotypic data collection. PC, MB, and GK designed the KASPar assays. KSG and GK wrote the manuscript and SB edited it. All authors read, commented, and approved the final manuscript.

## Conflict of Interest

The authors declare that the research was conducted in the absence of any commercial or financial relationships that could be construed as a potential conflict of interest.

## Publisher’s Note

All claims expressed in this article are solely those of the authors and do not necessarily represent those of their affiliated organizations, or those of the publisher, the editors and the reviewers. Any product that may be evaluated in this article, or claim that may be made by its manufacturer, is not guaranteed or endorsed by the publisher.

## Footnotes

1
http://www.ncbi.nlm.nih.gov/VecScreen/VecScreen.html

2
www.invitrogen.com/VectorNTI

## Supplementary Material

Supplementary File 1 Nucleotide sequences of *FAE1.1* (*B. rapa and B. juncea*) and *FAE1.2* (*B. nigra* and *B. juncea*) genes in the Fast Adaptive Shrinkage Threshold Algorithm (FASTA) format.

Supplementary Figure 1 Structure of *FAE1.1* gene of *B. rapa* genotypes **(A)** TL17 (high erucic acid) and **(B)** QR 2 (low erucic acid).

Supplementary Figure 2 Alignment of nucleotides and amino acids derived from gene sequences of *FAE1.1* genes from high (TL17) and low erucic acid (QR 2) genotypes of *B. rapa*.

Supplementary Figure 3 Structure of *FAE1.2* gene. **(A)** *B. nigra* (UP: high erucic acid; **B)**. *B. juncea* (RLC 3: low erucic acid).

Supplementary Figure 4 Alignment of nucleotides and amino acids derived from gene sequences of *FAE1.2* genes from high (UP) and low erucic acid (RLC 3) genotypes of *B. nigra and B. juncea*, respectively.

Supplementary Figure 5 Alignment of nucleotides of *FAE1.1* and *FAE1.2* genes of *B. juncea* (PBR91: high erucic acid and RLC 3: low erucic acid).

Supplementary Table 1 Source of diverse germplasm lines of *B. juncea*, *B. rapa*, and *B. nigra*.
