# Supplementary material for: Development and Validation of Kompetitive Allele-Specific PCR Assays for Erucic Acid Content in Indian Mustard [Brassica juncea (L.) Czern and Coss.]
Source: Front Plant Sci. 2021 Dec 15;12:738805. doi: 10.3389/fpls.2021.738805 (PMC8714676; doi:10.3389/fpls.2021.738805)
Supplement: Supplementary File 1 — Nucleotide sequences of FAE1.1 (B. rapa and B. juncea) and FAE1.2 (B. nigra and B. juncea) genes in the Fast Adaptive Shrinkage Threshold Algorithm (FASTA) format. [file Data_Sheet_1.PDF]

>FAE1.1_TL17

ATGACGTCCGTTAACGTAAAGCTCCTTTACCATTACGTCATAACCAACCTTTTCAACCTTTGCTTCTTTC
CGTTAACGGCGATCGTCGCCGGAAAAGCCTATCGGCTTACCATAGACGATCTTCACCACTTATACTATTC
CTATCTCCAACACAACCTCATAACCATCGCTCCACTCTTTGCCTTCACCGTTTTCGGTTCGGTTCTCTAC
ATCGCAACCCGGCCCAAACCGGTTTACCTCGTTGAGTACTCATGCTACCTTCCACCAACGCATTGTAGAT
CAAGTATCTCCAAGGTCATGGATATCTTTTATCAAGTAAGAAAAGCTGATCCTTCTCGGAACGGCACGTG
CGATGACTCGTCGTGGCTTGACTTCTTGAGGAAGATTCAAGAACGTTCAGGTCTAGGCGATGAAACTCAC
GGGCCCGAGGGGCTGCTTCAGGTCCCTCCCCGGAAGACTTTTGCGGCGGCGCGTGAAGAGACGGAGCAAG
TTATCATTGGTGCGCTAGAAAATCTATTCAAGAACACCAACGTTAGCCCTAAAGATATAGGTATACTTGT
GGTGAACTCAAGCATGTTTAATCCAACTCCGTCGCTCTCCGCGATGGTCGTTAACACTTTCAAGCTCCGA
AGCAACGTAAGAAGCTTTAACCTTGGTGGCATGGGTTGTAGTGCCGGCGTTATAGCCATTGATCTAGCAA
AGGACTTGTTGCATGTCCATAAAAATACGTATGCCCTTGTGGTGAGCACAGAGAACATCACTTATAACAT
TTACGCTGGTGATAATAGGTCCATGATGGTTTCAAATTGCTTGTTCCGTGTTGGTGGGGCCGCTATTTTG
CTCTCCAACAAGCCTGGAGATCGTAGACGGTCCAAGTACGAGCTAGTTCACACGGTTCGAACGCATACCG
GAGCTGACGACAAGTCTTTTCGTTGCGTGCAACAAGGAGACGATGAGAACGGCAAAACCGGAGTGAGTTT
GTCCAAGGACATAACCGATGTTGCTGGTCGAACGGTTAAGAAAAACATAGCAACGTTGGGTCCGTTGATT
CTTCCGTTAAGCGAGAAACTTCTTTTTTTCGTTACCTTCATGGGCAAGAAACTTTTCAAAGATAAAATCA
AACATTACTACGTCCCGGATTTCAAACTTGCTATTGACCATTTTTGTATACATGCCGGAGGCAGAGCCGT
GATTGATGTGCTAGAGAAGAACCTAGCCCTAGCACCGATCGATGTAGAGGCATCAAGATCAACGTTACAT
AGATTTGGAAACACTTCATCTAGCTCAATATGGTATGAGTTGGCATACATAGAAGCAAAAGGAAGGATGA
AGAAAGGTAATAAAGTTTGGCAGATTGCTTTAGGGTCAGGCTTTAAGTGTAACAGTGCAGTTTGGGTGGC
TCTAAACAATGTCAAAGCTTCGACAAATAGTCCTTGGGAACACTGCATCGACAGATACCCGGTCAAAATT
GATTCTGATTCAGGTAAGTCAGAGACTCGTGTCCAAAACGGTCGGTCCTAA

>FAE1.1_QR2

ATGACGTCCGTTAACGTAAAGCTCCTTTACCATTACGTCATAACCAACCTTTTCAACCTTTGCTTCTTTC
CGTTAACGGCGATCGTCGCCGGAAAAGCCTATCGGCTTACCATAGACGATCTTCACCACTTATACTATTC
CTATCTCCAACACAACCTCATAACCATCGCTCCACTCTTTGCCTTCACCGTTTTCGGTTCGGTTCTCTAC
ATCGCAACCCGGCCCAAACCGGTTTACCTCGTTGAGTACTCATGCTACCTTCCACCAACGCATTGTAGAT
CAAGTATCTCCAAGGTCATGGATATCTTTTATCAAGTAAGAAAAGCTGATCCTTCTCGGAACGGCACGTG
CGATGACTCGTCGTGGCTTGACTTCTTGAGGAAGATTCAAGAACGTTCAGGTCTAGGCGATGAAACTCAC
GGGCCCGAGGGGCTGCTTCAGGTCCCTCCCCGGAAGACTTTTGCGGCGGCGCGTGAAGAGACGGAGCAAG
TTATCATTGGTGCGCTTGAAAATCTATTCAAGAACACCAACGTTAACCCTAAAGATATAGGTATACTTGT
GGTGAACTCAAGCATGTTTAATCCAACTCCATCGCTCTCCGCGATGGTCGTTAACACTTTCAAGCTCCGA
AGCAACGTAAGAAGCTTTAACCTTGGTGGCATGGGTTGTAGTGCTGGCGTTATAGCCATTGATCTGGCAA
AGGACTTGTTGCATGTCCATAAAAATACGTATGCTCTTGTGGTGAGCACAGAGAACATCACTTATAACAT
TTACGCTGGTGATAATAGGTCCATGATGGTTTCAAATTGCTTGTTCCGTGTTGGTGGGGCCGCTATTTTG
CTCTCCAACAAGCCTGGAGATCGTAGACGGTCCAAGTACGAGCTAGTTCACACGGTTCGAACGCATACCG
GAGCTGACGACAAGTCTTTTCGTTGCGTGCAACAAGGAGACGATGAGAACGGCAAAATCGGAGTGAGTTT
GTCCAAGGACATAACCGATGTTGCTGGTCGAACGGTTAAGAAAAACATAGCAACGTTGGGTCCGTTGATT
CTTCCGTTAAGCGAGAAACTTCTTTTTTTCGTTACCTTCATGGGCAAGAAACTTTTCAAAGATAAAATCA

AACATTACTACGTCCCGGATTTCAAACTTGCTATTGACCATTTTTGTATACATGCCGGAGGCAGAGCCGT
GATTGATGTGCTAGAGAAGAACCTAGCCCTAGCACCGATCGATGTAGAGGCATCAAGATCAACGTTACAT
AGATCTGGAAACACTTCATCTAGCTCAATATGGTATGAGTTGGCATACATAGAAGCAAAAGGAAGGATGA
AGAAAGGTAATAAAGTTTGGCAGATTGCTTTAGGGTCAGGCTTTAAGTGTAACAGTGCAGTTTGGGTGGC
TCTAAACAATGTCAAAGCTTCGACAAATAGTCCTTGGGAACACTGCATCGACAGATACCCGGTCAAAATT
GATTCTGATTCAGGTAAGTCAGAGACTCGTGTCCAAAACGGTCGGTCCTAA

>FAE1.2_UP

ATGACGTCCGTTAACGTAAAGCTCCTTTACCATTACGTCATAACCAACTTTTTCAACCTTTGCTTCTTCC
CGTTAACGGCGATCGTCGCCGGAAAAGCCTCTCGGCTTACCATAGACGATCTTCACCACTTATACTATTC
CTATCTCCAACACAACCTCATAACCATCGCTCCACTCTTTGCCTTCACCGTTTTCGGTTCGGTTCTCTAC
ATCGCAACCCGGCCCAAACCGGTTTACCTCGTTGAGTACTCATGCTACCTTCCACCAACGCATTGCAGAT
CAAGTATCTCCAAGGTCATGGATATATTTTATCAAGTAAGAAAAGCTGATCCTTCTCGGAACGGGACGTG
CGATGACTCGTCGTGGCTTGACTTCTTGAGGAAGATTCAAGAACGTTCGGGTCTAGGCGATGAAACTTAC
GGGCCAGAGGGGCTGCTTCAGGTCCCTCCTCGGAAGACTTTCGCGGCGGCGCGTGAAGAGACGGAGCAAG
TAATCATCGGTGCGCTTAAAAATCTATTCGAGAACACCAAAGTTAACCCTAAAGATATAGGTATACTCGT
GGTGAACTCAAGCATGTTTAATCCAACTCCTTCACTCTCCGCGATGGTCGTGAACACTTTCAAGCTCCGA
AGCAACGTAAGAAGCTTTAATCTCGGTGGCATGGGTTGTAGTGCTGGTGTTATAGCCATTGACCTGGCTA
AGGACTTGTTGCATGTCCATAAAAACACGTATGCTCTTGTGGTGAGCACAGAGAACATCACTTATAACAT
TTACGCTGGTGATAATAGGTCCATGATGGTTTCGAACTGCTTGTTCCGTGTTGGTGGGGCAGCTATTTTG
CTCTCCAACAAGCCTAGAGATCGTAGACGGTCCAAGTACGAGCTAGTTCACACGGTTCGAACGCATACCG
GAGCTGACGACAAGTCTTTTAGATGCGTGCAACAAGGAGACGATGAGAACGGCAAAACCGGAGTGAGTTT
ATCTAAGGACATAACCGATGTTGCTGGTCGAACGGTCAAGAAAAACATAGCAACGTTAGGTCCGTTGATT
CTTCCTTTAAGCGAGAAACTTCTCTTTTTCGTTACATTCATGGCCAAAAAACTTTTCAAAGACAAAGTTA
AGCATTACTACGTCCCGGACTTCAAGCTTGCTGTTGACCATTTTTGTATACATGCTGGAGGCAGAGCCGT
GATCGATGTGCTCGAGAAGAACCTAGGCCTAGCACCGATCGATGTAGAGGCATCAAGATCAACGTTACAT
AGATTTGGTAACACTTCATCTAGCTCGATATGGTATGAATTGGCATACATAGAAGCAAAAGGAAGGATGA
AGAAAGGTAATAAAGTTTGGCAGATTGCTTTAGGGTCAGGGTTTAAGTGTAACAGTGCGGTTTGGGTGGC
TCTAAGCAATGTCAAGGCTTCGACAAATAGTCCTTGGGAACATTGCATCGACAGATACCCGGTTAAAATT
GATTCTGATTCAGCTAAGTCAGAGGTTCGTGTCCAAAACGGTCGGTCCTAA

> FAE1.1_PBR91

ATGACGTCCGTTAACGTAAAGCTCCTTTACCATTACGTCATAACCAACCCTTTCAACCTTTGCTTCTTTC
CGTTAACGGCGATCGTCGCCGGAAAAGCCTATCGGCTTACCATAGACGATCTTCACCACTTATACTATTC
CTATCTCCAACACAACCTCATAACCATCGCTCCACTCTTTGCCTTCACCGTTTTCGGTTCGGTTCTCTAC
ATCGCAACCCGGCCCAAACCGGTTTACCTCGTTGAGTACTCATGCTACCTTCCACCAACGCATTGTAGAT
CAAGTATCTCCAAGGTCATGGATATCTTTTATCAAGTAAGAAAAGCTGATCCTTCTCGGAACGGCACGTG
CGATGACTCGTCGTGGCTTGACTTCTTGAGGAAGATTCAAGAACGTTCAGGTCTAGGCGATGAAACTCAC
GGGCCCGAGGGGCTGCTTCAGGTCCCTCCCCGGAAGACTTTTGCGGCGGCGCGTGAAGAGACGGAGCAAG
TTATCATTGGTGCGCTAGAAAATCTATTCAAGAACACCAACGTTAACCCTAAAGATATAGGTATACTTGT
GGTGAACTCAAGCATGTTTAATCCAACTCCGTCGCTCTCCGCGATGGTCGTTAACACTTTCAAGCTCCGA

AGCAACGTAAGAAGCTTTAACCTTGGTAGCATGGGTTGTAGTGCCGGCGTTATAGCCATTGATCTAGCAA
AGGACTTGTTGCATGTCCATAAAAATACGTATGCCCTTGTGGTGAGCACAGAGAACATCACTTATAACAT
TTACGCTGGTGATAATAGGTCCATGATGGTTTCAAATTGCTTGTTCCGTGTTGGTGGGGCCGCTATTTTG
CTCTCCAACAAGCCTGGAGATCGTAGACGGTCCAAGTACGAGCTAGTTCACACGGTTCGAACGCATACCG
GAGCTGACGACAAGTCTTTTCGTTGCGTGCAACAAGGAGACGATGAGAACGGCAAAACCGGAGTGAGTTT
GTCCAAGGACATAACCGATGTTGCTGGTCGAACGGTTAAGAAAAACATAGCAACGTTGGGTCCGTTGATT
CTTCCGTTAAGCGAGAAACTTCTTTTTTTCGTTACCTTCATGGGCAAGAAACTTTTCAAAGATAAAATCA
AACATTACTACGTCCCGGATTTCAAACTTGCTATTGACCATTTTTGTATACATGCCGGAGGCAGAGCCGT
GATTGATGTGCTAGAGAAGAACCTAGCCCTAGCACCGATCGATGTAGAGGCATCAAGATCAACGTTACAT
AGATTTGGAAACACTTCATCTAGCTCAATATGGTATGAGTTGGCATACATAGAAGCAAAAGGAAGGATGA
AGAAAGGTAATAAAGTTTGGCAGATTGCTTTAGGGTCAGGCTTTAAGTGTAACAGTGCAGTTTGGGTGGC
TCTAAACAATGTCAAAGCTTCGACAAATAGTCCTTGGGAACACTGCATCGACAGATACCCGGTCAAAATT
GATTCTGATTCAGGTAAGTCAGAGACTCGTGTCCAAAACGGTCGGTCCTAA

> FAE1.2_PBR91

ATGACGTCCGTTAACGTAAAGCTCCTTTACCATTACGTCATAACCAACTTTTTCAACCTTTGCTTCTTCC
CGTTAACGGCGATCGTCGCCGGAAAAGCCTCTCGGCTTACCATAGACGATCTTCACCACTTATACTATTC
CTATCTCCAACACAACCTCATAACCATCGCTCCACTCTTTGCCTTCACCGTTTTCGGTTCGGTTCTCTAC
ATCGCAACCCGGCCCAAACCGGTTTACCTCGTTGAGTACTCATGCTACCTTCCACCAACGCATTGCAGAT
CAAGTATCTCCAAGGTCATGGATATATTTTATCAAGTAAGAAAAGCTGATCCTTCTCGGAACGGGACGTG
CGATGACTCGTCGTGGCTTGACTTCTTGAGGAAGATTCAAGAACGTTCGGGTCTAGGCGATGAAACTTAC
GGGCCAGAGGGGCTGCTTCAGGTCCCTCCTCGGAAGACTTTCGCGGCGGCGCGTGAAGAGACGGAGCAAG
TAATCATCGGTGCGCTTAAAAATCTATTCGAGAACACCAAAGTTAACCCTAAAGATATAGGTATACTCGT
GGTGAACTCAAGCATGTTTAATCCAACTCCTTCACTCTCCGCGATGGTCGTGAACACTTTCAAGCTCCGA
AGCAACGTAAGAAGCTTTAATCTCGGTGGCATGGGTTGTAGTGCTGGTGTTATAGCCATTGACCTGGCTAA
GGACTTGTTGCATGTCCATAAAAACACGTATGCTCTTGTGGTGAGCACAGAGAACATCACTTATAACATTT
ACGCTGGTGATAATAGGTCCATGATGGTTTCGAACTGCTTGTTCCGTGTTGGTGGGGCAGCTATTTTGCTCT
CCAACAAGCCTAGAGATCGTAGACGGTCCAAGTACGAGCTAGTTCACACGGTTCGAACGCATACCGGAGC
TGACGACAAGTCTTTTAGATGCGTGCAACAAGGAGACGATGAGAACGGCAAAACCGGAGTGAGTTTATCT
AAGGACATAACCGATGTTGCTGGTCGAACGGTCAAGAAAAACATAGCAACGTTAGGTCCGTTGATTCTTC
CTTTAAGCGAGAAACTTCTCTTTTTCGTTACATTCATGGCCAAAAAACTTTTCAAAGACAAAGTTAAGCA
TTACTACGTCCCGGACTTCAAGCTTGCTGTTGACCATTTTTGTATACATGCTGGAGGCAGAGCCGTGATC
GATGTGCTCGAGAAGAACCTAGGCCTAGCACCGATCGATGTAGAGGCATCAAGATCAACGTTACATAGAT
TTGGTAACACTTCATCTAGCTCGATATGGTATGAATTGGCATACATAGAAGCAAAAGGAAGGATGAAGAA
AGGTAATAAAGTTTGGCAGATTGCTTTAGGGTCAGGGTTTAAGTGTAACAGTGCGGTTTGGGTGGCTCTA
AGCAATGTCAAGGCTTCGACAAATAGTCCTTGGGAACATTGCATCGACAGATACCCGGTTAAAATTGATT
CTGATTCAGCTAAGTCAGAGGTTCGTGTCCAAAACGGTCGGTCCTAA

>FAE1.1_RLC3

ATGACGTCCGTTAACGTAAAGCTCCTTTACCATTACGTCATAACCAACCTTTTCAACCTTTGCTTCTTTCCGTTAACG
GCGATCGTCGCCGGAAAAGCCTATCGGCTTACCATAGACGATCTTCACCACTTATACTATTCCTATCTCCAACACAA

CCTCATAACCATCGCTCCACTCTTTGCCTTCACCGTTTTCGGTTCGGTTCTCTACATCGCAACCCGGCCCAAACCGGT
TTACCTCGTTGAGTACTCATGCTACCTTCCACCAACGCATTGTAGATCAAGTATCTCCAAGGTCATGGATATCTTTTA
TCAAGTAAGAAAAGCTGATCCTTCTCGGAACGGCACGTGCGATGACTCGTCGTGGCTTGACTTCTTGAGGAAGATT
CAAGAACGTTCAGGTCTAGGCGATGAAACTCACGGGCCCGAGGGGCTGCTTCAGGTCCCTCCCCGGAAGACTTTT
GCGGCGGCGCGTGAAGAGACGGAGCAAGTTATCATTGGTGCGCTAGAAAATCTATTCAAGAACACCAACGTTAAC
CCTAAAGATATAGGTATACTTGTGGTGAACTCAAGCATGTTTAATCCAACTCCATCGCTCTCCGCGATGGTCGTTAA
CACTTTCAAGCTCCGAAGCAACGTAAGAAGCTTTAACCTTGGTGGCATGGGTTGTAGTGCCGGCGTTATAGCCATT
GATCTAGCAAAGGACTTGTTGCATGTCCATAAAAATACGTATGCTCTTGTGGTGAGCACAGAGAACATCACTTATA
ACATTTACGCTGGTGATAATAGGTCCATGATGGTTTCAAATTGCTTGTTCCGTGTTGGTGGGGCCGCTATTTTGCTC
TCCAACAAGCCTGGAGATCGTAGACGGTCCAAGTACGAGCTAGTTCACACGGTTCGAACGCATACCGGAGCTGAC
GACAAGTCTTTTCGTTGCGTGCAACAAGGAGACGATGAGAACGGCAAAATCGGAGTGAGTTTGTCCAAGGACATA
ACCGATGTTGCTGGTCGAACGGTTAAGAAAAACATAGCAACGTTGGGTCCGTTGATTCTTCCGTTAAGCGAGAAA
CTTCTTTTTTTCGTTACCTTCATGGGCAAGAAACTTTTCAAAGATAAAATCAAACATTACTACGTCCCGGATTTCAAA
CTTGCTATTGACCATTTTTGTATACATGCCGGAGGCAGAGCCGTGATTGATGTGCTAGAGAAGAACCTAGCCCTAG
CACCGATCGATGTAGAGGCATCAAGATCAACGTTACATAGATCTGGAAACACTTCATCTAGCTCAATATGGTATGA
GTTGGCATACATAGAAGCAAAAGGAAGGATGAAGAAAGGTAATAAAGTTTGGCAGATTGCTTTAGGGTCAGGCT
TTAAGTGTAACAGTGCAGTTTGGGTGGCTCTAAACAATGTCAAAGCTTCGACAAATAGTCCTTGGGAACACTGCAT
CGACAGATACCCGGTCAAAATTGATTCTGATTCAGGTAAGTCAGAGACTCGTGTCCAAAACGGTCGGTCCTAA

>FAE1.2_RLC3

ATGACGTCCGTTAACGTAAAGCTCCTTTACCATTACGTCATAACCAACCTTTTCAACCTTTGCTTCTTCCCGTTAACG
GCGATCGTCGCCGGAAAAGCCTCTCGGCTTACCATAGACGATCTTCACCACTTATACTATTCCTATCTCCAACACAA
CCTCATAACCATCGCTCCACTCTTTGCCTTCACCGTTTTCGGTTCGGTTCTCTACATCGCAACCCGGCCCAAACCGGT
TTATCTCGTTGAGTACTCATGCTACCTTCCACCAACGCATTGCAGATCAAGTATCTCCAAGGTCATGGATATATTTTA
TCAAGTAAGAAAAGCTGATCCTTCTCGGAACGGGACGTGCGATGACTCGTCGTGGCTTGACTTCTTGAGGAAGAT
TCAAGAACGTTCGGGTCTAGGCGATGAAACTTACGGGCCAGAGGGGCTGCTTCAGGTCCCTCCTCGGAAGACTTT
CGCGGCGGCGCGTGAAGAGACGGAGCAAGTAATCATCGGTGCGCTTAAAAATCTATTCGAGAACACCAAAGTTA
ACCCTAAAGATATAGGTATACTCGTGGTGAACTCAAGCATGTTTAATCCAACTCCTTCACTCTCCGCGATGGTCGTG
AACACTTTCAAGCTCCGAAGCAACGTAAGAAGCTTTAATCTCGGTGGCATGGGTTGTAGTGCTGGTGTTATAGCCA
TTGACCTGGCTAAGGACTTGTTGCATGTCCATAAAAACACGTATGCTCTTGTGGTGAGCACAGAGAACATCACTTA
TAACATTTACGCTGGTGATAATAGGTCCATGATGGTTTCGAACTGCTTGTTCCGTGTTGGTGGGGCAGCTATTTTG
CTCTCCAACAAGCCTAGAGATCGTAGACGGTCCAAGTACGAGCTAGTTCACACGGTTCGAACGCATACCGGAGCT
GACGACAAGTCTTTTAGATGCGTGCAACAAGGAGACGATGAGAACGGCAAAACCGGAGTGAGTTTATCTAAGGA
CATAACCGATGTTGCTGGTCGAACGGTCAAGAAAAACATAGCAACGTTAGGTCCGTTGATTCTTCCTTTAAGCGAG
AAACTTCTCTTTTTCGTTACATTCATGGCCAAAAAACTTTTCAAAGACAAAGTTAAGCATTACTACGTCCCGGACTTC
AAGCTTGCTGTTGACCATTTTTGTATACATGCTGGAGGCAGAGCCGTGATCGATGTGCTCGAGAAGAACCTAGGC
CTAGCACCGATCGATGTAGAGGCATCAAGATCAACGTTACATAGATTTGGTAACACTTCATCTAGCTCGATATGGT
ATGAATTGGCATACATAGAAGCAAAAGGAAGGATGAAGAAAGGTAATAAAGTTTGGCAGATTGCTTTAGGGTCA
GGGTTTAAGTGTAACAGTGCGGTTTGGGTGGCTCTAAGCAATGTCAAGGCTTCGACAAATAGTCCTTGGGAACAT
TGCATCGACAGATACCCGGTTAAAATTGATTCAGATTCAGCTAAGTCAGAGGTTCGTGTCCAAAACGGTCGGTCCT
GA
