# Supplementary figures and images for: Development and Validation of Kompetitive Allele-Specific PCR Assays for Erucic Acid Content in Indian Mustard [Brassica juncea (L.) Czern and Coss.]
Source: Front Plant Sci. 2021 Dec 15;12:738805. doi: 10.3389/fpls.2021.738805 (PMC8714676; doi:10.3389/fpls.2021.738805)

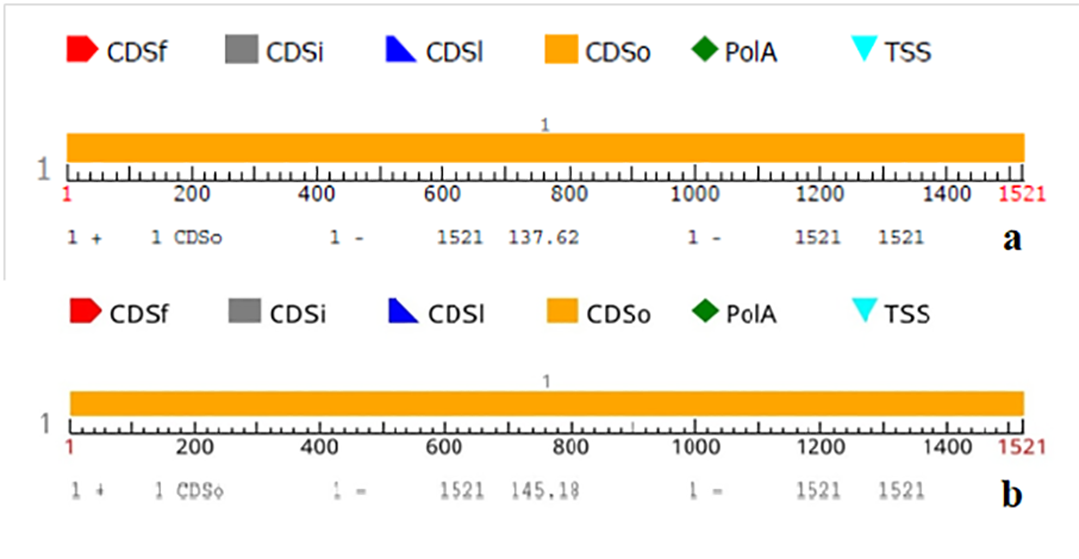

Supplement: Supplementary Figure 1 — Structure of FAE1.1 gene of B. rapa genotypes (A) TL17 (high erucic acid) and (B) QR 2 (low erucic acid). [file Image_1.TIF]

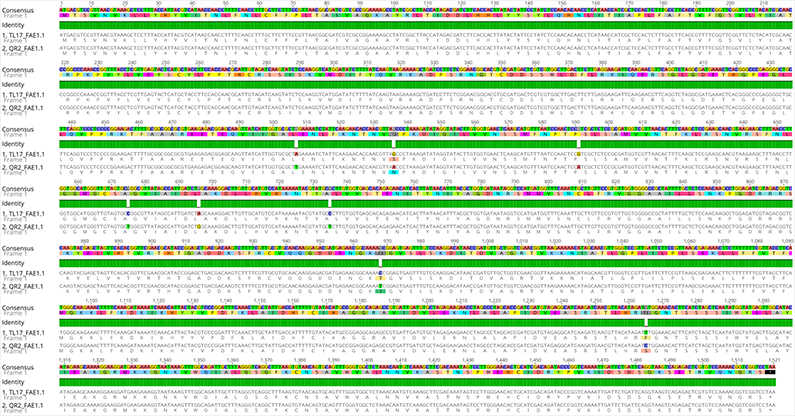

Supplement: Supplementary Figure 2 — Alignment of nucleotides and amino acids derived from gene sequences of FAE1.1 genes from high (TL17) and low erucic acid (QR 2) genotypes of B. rapa. [file Image_2.TIFF]

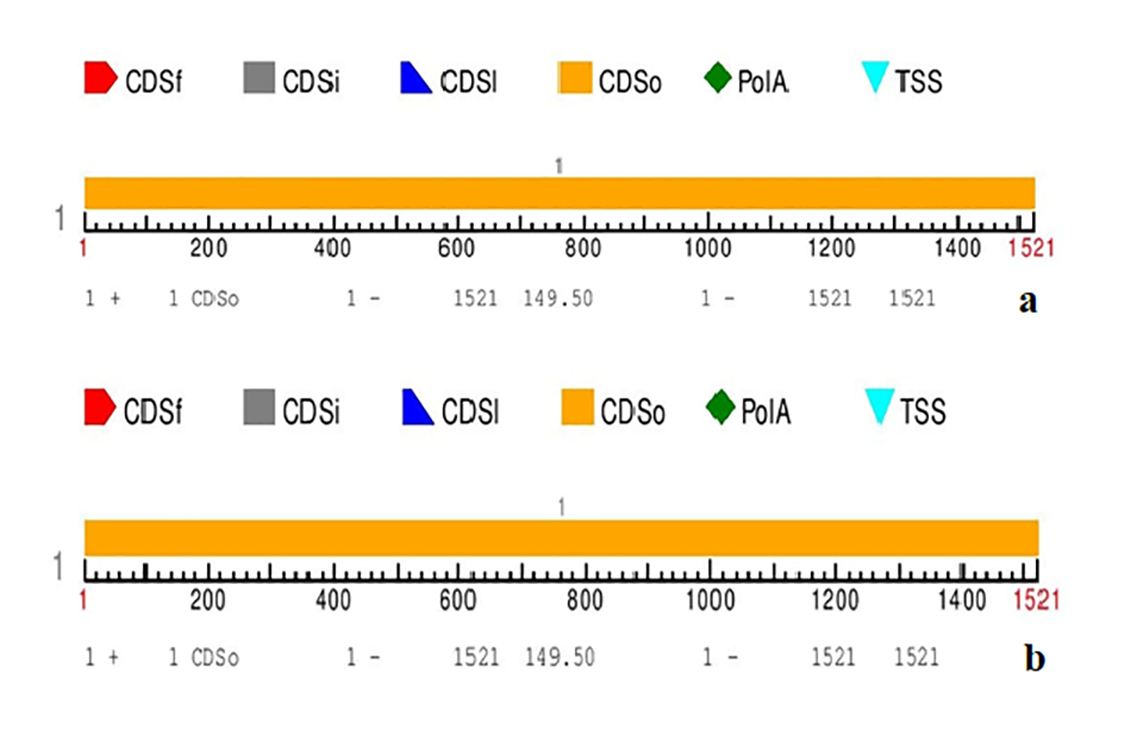

Supplement: Supplementary Figure 3 — Structure of FAE1.2 gene. (A) B. nigra (UP: high erucic acid; B). B. juncea (RLC 3: low erucic acid). [file Image_3.TIF]

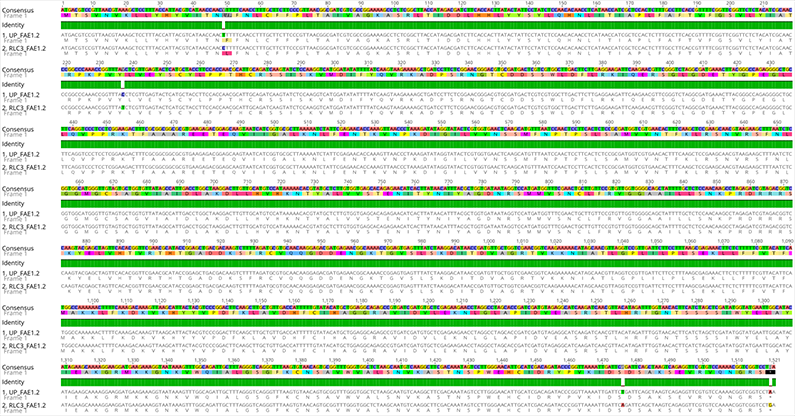

Supplement: Supplementary Figure 4 — Alignment of nucleotides and amino acids derived from gene sequences of FAE1.2 genes from high (UP) and low erucic acid (RLC 3) genotypes of B. nigra and B. juncea, respectively. [file Image_4.TIFF]

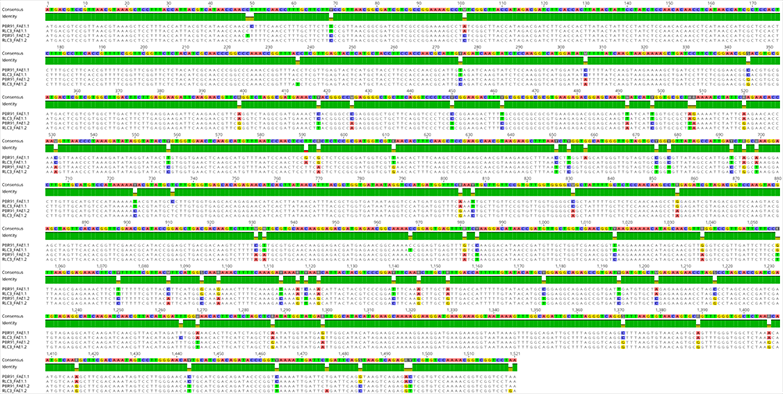

Supplement: Supplementary Figure 5 — Alignment of nucleotides of FAE1.1 and FAE1.2 genes of B. juncea (PBR91: high erucic acid and RLC 3: low erucic acid). [file Image_5.TIFF]
