# Supplementary material for: Development and Validation of Kompetitive Allele-Specific PCR Assays for Erucic Acid Content in Indian Mustard [Brassica juncea (L.) Czern and Coss.]
Source: Front Plant Sci. 2021 Dec 15;12:738805. doi: 10.3389/fpls.2021.738805 (PMC8714676; doi:10.3389/fpls.2021.738805)
Supplement: Supplementary Table 1 — Source of diverse germplasm lines of B. juncea, B. rapa, and B. nigra. [file Table_1.DOCX]

**Additional File 8: Table S1. Source of diverse germplasm lines of *B. juncea, B. rapa* and *B. nigra***

| **Genotype** | **Origin** | **Source** |
| --- | --- | --- |
| ALM 115 | Indian | PAU, Ludhiana |
| ELM 303 | Indian | PAU, Ludhiana |
| JLM 102 | Indian | PAU, Ludhiana |
| JM 06013 | Australian | PAU, Ludhiana |
| PBR 210 | Indian | PAU, Ludhiana |
| PBR 357 | Indian | PAU, Ludhiana |
| PBR 378 | Indian | PAU, Ludhiana |
| PBR 91 | Indian | PAU, Ludhiana |
| QR1 | Indian | PAU, Ludhiana |
| RL 1359 | Indian | PAU, Ludhiana |
| RLC-1 | Indian | PAU, Ludhiana |
| RLC-2 | Indian | PAU, Ludhiana |
| RLC-3 | Indian | PAU, Ludhiana |
| RLM 619 | Indian | PAU, Ludhiana |
| TL-17 | Indian | PAU, Ludhiana |
| U.P | Indian | PAU, Ludhiana |
| DRMRIJ-31 | Indian | DRMR, Bharatpur |
| NRCDR-02 | Indian | DRMR, Bharatpur |
| PDZ-1 | Indian | IARI, New Delhi |
| PM 24 | Indian | IARI, New Delhi |
| PM 30 | Indian | IARI, New Delhi |
| Pusa Bold | Indian | IARI, New Delhi |
| RH 0749 | Indian | CCSHAU, Hisar |
| RH 8812 | Indian | CCSHAU, Hisar |
| EC 597325 | Sweden | NBPGR, New Delhi |
| HEERA | Europe | DU, New Delhi |
| CN 113799 | ---- | Plant Gene Resources, Canada |
| Donskaja | Europe | Gene Bank, Canada |
| CBJ 001 | China | HZAU, China |

PAU: Punjab Agricultural University

DRMR: Directorate of Rapeseed Mustard Research

IARI: Indian Agricultural Research Institute

CCSHAU: Chaudhary Charan Singh Haryana Agricultural University

NBPGR: National Bureau of Plant Genetic Resources

DU: Delhi University

HZAU: Huazhong Agricultural University
